# Study on Preparation of Calcium-Based Modified Coal Gangue and Its Adsorption Dye Characteristics

**DOI:** 10.3390/molecules29102183

**Published:** 2024-05-07

**Authors:** Yihan Wang, Yanrong Dong, Junli Shao, Zilong Zhao, Hongyu Zhai

**Affiliations:** 1 College of Civil Engineering, Liaoning Technical University, Fuxin 123000, China; wyh18842017767@126.com (Y.W.); 18265374325@163.com (H.Z.); 2 College of Mechanics and Engineering, Liaoning Technical University, Fuxin 123000, China; 3 College of Mining, Liaoning Technical University, Fuxin 123000, China; 15836906028@163.com

**Keywords:** adsorption, calcium-based, coal gangue, dye, characteristic

## Abstract

Efficient and thorough treatment of dye wastewater is essential to achieve ecological harmony. In this study, a new type of calcium-based modified coal gangue (Ca-CG) was prepared by using solid waste coal gangue as raw material and a CaCl_2_ modifier, which was used for the removal of malachite green, methylene blue, crystal violet, methyl violet and other dyes in water. When the dosage of Ca-CG was 1–5 g/L, the dosage of Ca-CG was the main factor affecting the dye adsorption effect. The adsorption effects of Ca-CG on four dyes were as follows: malachite green > crystal violet > methylene blue > methyl violet. Kinetics, isotherms and thermodynamic analysis showed that the adsorption of malachite green, methyl blue, crystal violet and methyl violet by Ca-CG fitted the second-order kinetic model, and adsorption with chemical reaction is the main process. The adsorption of four dyes by Ca-CG conformed to the Freundlich model, which is dominated by multi-molecular layer adsorption, and the adsorption was easy to carry out. The adsorption process of Ca-CG on the four dyes was spontaneous. The results of FTIR, XRD and SEM showed that the calcium-based materials such as lipscombite and dolomite were the key to the adsorption of malachite green by Ca-CG, and the main mechanisms for the adsorption of malachite green by Ca-CG are surface precipitation, electrostatic action, and chelation reaction. Ca-CG adsorption has great potential for the removal of dye wastewater.

## 1. Introduction

With the rapid development of the textile industry, printing industry, and laboratory research (Enhanced Adsorptive Removal of Methyl Orange and Methylene Blue from Aqueous Solution by Alkali-Activated Multiwalled Carbon Nanotubes), the pollution problem of difficult-to-degrade dye wastewater is becoming increasingly serious [1]. About 280,000 tons of dye wastewater is discharged worldwide, accounting for about 80% of total industrial wastewater discharge [2]. To achieve ecological harmony, how to efficiently and thoroughly treat dye wastewater is one of the key issues that must be considered [3,4]. Dyes usually have aromatic structures, resulting in strong oxidation resistance and light stability of dye wastewater, which is difficult to remove by traditional wastewater treatment technology. Malachite green, methylene blue, crystal violet and methyl violet are common organic dyes in dye wastewater. They have certain biological toxicity and are easy to have harmful effects on the natural environment and human health [5]. In the treatment of dye wastewater, conventional water treatment methods such as chemical and biological methods have limitations such as incomplete decomposition, high operational costs, poor decolorization effect, secondary pollution generation and so on. Additionally, there are reports on the use of ozone oxidation, electrolytic oxidation, and photocatalytic oxidation methods for treating dye wastewater. For instance, Xu et al. [6] developed a process involving O_3_ + MnOx/GAC + H_2_O_2_, which achieved a removal rate of over 80% for organic halides in dye wastewater. However, ozone oxidation may exhibit incomplete removal of organic pollutants from dyes and may result in the formation of intermediate products, making this method more expensive [7]. Seema et al. [8] reported that under conditions of pH 6.0 and 30 °C in a 254 nm UV photocatalytic oxidation process, 90% of red blood cell dye (50 ppm) was removed within 6 h. Under conditions of pH 6.0 and a NaCl electrolyte concentration of 0.1 mol/L, the degradation rate of red blood cell dye (50 ppm) reached 99.61% after 10 min of electrolysis [8]. The results indicated that the electrolytic oxidation process was more effective in removing dyes in a shorter period compared to photocatalytic oxidation [8]. However, the electrolytic oxidation process can produce toxic substances and is less effective for hydrophobic materials, making its application relatively challenging [7]. According to reports, adsorption is the simplest and most effective method for treating dye wastewater [9]. At present, the most common adsorbent for the treatment of dye wastewater is activated carbon [10,11]. However, the obvious shortage of activated carbon is the high cost of production, and the high market price is not conducive to large-scale application [12]. Therefore, finding cheaper adsorption materials to replace activated carbon can effectively reduce the cost of treating dye wastewater. In this regard, a solid waste material coal gangue can be used in mining areas. A large number of scholars have proved that coal gangue can be used as a wastewater remediation material. But the adsorption capacity of coal gangue is limited [13]. Interestingly, coal gangue processed by chemistry and calcination can improve the adsorption performance. Shang [14] found that the adsorption capacity of Pb^2+^, Cd^2+^ and Hg^2+^ by thiol-modified coal gangue could reach 332.8 mg/g, 110.4 mg/g and 179.2 mg/g, respectively. Mohammadi [15] found that the adsorption capacity of Zn^2+^ and Mn^2+^ on iron oxide-modified coal gangue can reach 42.10 mg/g and 30.12 mg/g. Qiu B. [16] found that the adsorption of PO_4_^3−^ by biochar-processed coal gangue can reach 3.20 mg/g. Zhou [17] found that the porous ceramic microspheres prepared from coal gangue had removal capacities of 1.04 mg/g for cationic red dye and 2.17 mg/g for cationic blue dye. Haodong [18] found that coal gangue and peanut shell were calcined together to prepare a composite adsorbent, and the adsorption of methylene blue could reach 0.954 mg/g. The above study found that coal gangue can be used as a dye adsorbent. At the same time, chemical modification and calcination can improve the adsorption of coal gangue. Calcined coal gangue needs to strictly control the temperature, and the cost of preparing composite adsorption materials is high. Therefore, chemical modification of coal gangue is a better method to improve the adsorption performance of coal gangue. The adsorption capacity of calcined coal gangue for dyes is limited. Above all, chemical modification of coal gangue is a better method to enhance its adsorption performance. The above chemically modified coal gangue materials are mostly used in the field of heavy metal and PO_4_^3−^ treatment, and there are relatively few studies on the adsorption of dyes by chemically modified coal gangue.

Chemically modified Ca-doped adsorbent can improve the dye adsorption capacity of the adsorbent [19]. He et al. [19] prepared spherical magnetic calcium-modified chitosan microparticles with CaCl_2_ as raw material, and the adsorption effect of the modified material on methylene blue (MB) can reach 350 mg/g. An et al. [20] prepared a new calcium-based adsorbent CaFe_2_O_4_, which had a high affinity for congo red, and the removal rate of congo red dye was 96.1%. Behzat B. et al. [21] found that when 0.2 g of calcium peroxide was combined with 0.04 g of magnetite powdered activated carbon, the removal rate of reactive blue 21 reached 99.44%. Estevan D. Cruz [22] fixed Ca/double hydroxide on biochar by co-precipitation method, and the material had a good adsorption effect on congo red. Pengjie W. [23] reported that calcium doping can significantly change the morphology of the adsorbent material and improve the adsorption performance. In particular, the synthesized calcium-based magnetic biochar can efficiently adsorb malachite green dye. In summary, calcium-based modification can improve the adsorption performance of the adsorbent for dyes. However, the existing research lacks focus on the adsorption of dyes by calcium-based modified coal gangue. Compared with the above adsorbents, coal gangue is a kind of solid waste in mining areas. Using coal gangue as raw material and employing calcium-based modification techniques to enhance its dye adsorption performance can effectively increase the utilization rate of coal gangue and alleviate the problem of gangue accumulation. Moreover, developing a new type of dye adsorbent material enables the treatment of wastewater with waste, which has significant implications for environmentally friendly development.

Therefore, to address the issues of severe dye wastewater pollution and low adsorption capacity of coal gangue, the characteristics of enhancing adsorbent adsorption capacity through Ca modification are considered. In this paper, a new adsorbent calcium-based modified coal gangue (Ca-CG) was prepared by an ultrasonic method using coal gangue and CaCl_2_ as raw materials. Based on the adsorption of malachite green, methylene blue, crystal violet and methyl violet by Ca-CG, the characteristics of adsorption of malachite green, methylene blue, crystal violet and methyl violet by Ca-CG were analyzed by adsorption kinetics, adsorption isotherms and adsorption thermodynamics. At the same time, combined with the results of SEM, XRD and FTIR, the microscopic mechanism of Ca-CG-adsorbing dyes was revealed. Compared to coal gangue, Ca-CG demonstrates a higher adsorption capacity for dye wastewater. This technology provides a reference for the utilization of coal gangue and the remediation of dye wastewater pollution.

## 2. Results and Discussion

### 2.1. Experimental Results of Different Ca:CG Ratios of Ca-CG on Dye Removal Efficiency

**Figure 1:**
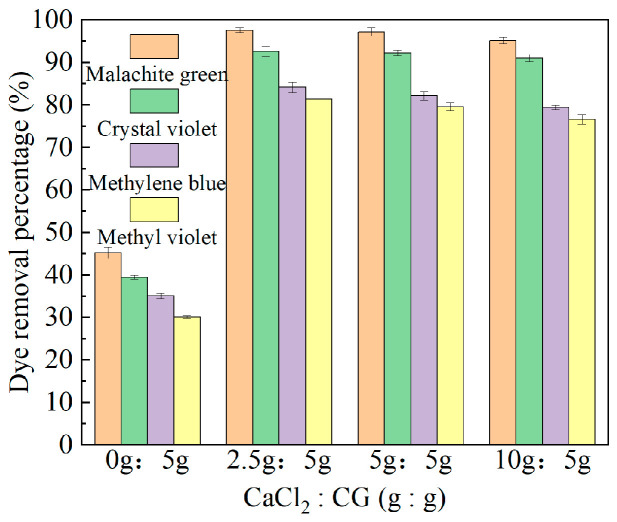
The removal efficiency of dyes by different Ca:CG Ca-CG samples. (Dye initial concentration: 200 mg/L, adsorbent dosage: 5 g/L, temperature: 298.15 K, agitation speed: 150 r/min, adsorption time: 150 min).

CaCl_2_/coal gangue ratios were 0:5 g, 2.5:5 g, 5:5 g, and 10:5 g; different Ca:CG Ca-CG samples were prepared. These Ca-CG samples were then used to adsorb malachite green, crystal violet, methylene blue, and methyl violet, as shown in Figure 1. From Figure 1, it can be observed that when the CaCl_2_/coal gangue ratio was 0:5 g, the removal rates of malachite green, crystal violet, methylene blue, and methyl violet by coal gangue were only 45.24%, 39.49%, 35.09%, and 30.15%, respectively. However, Ca-CG prepared with CaCl_2_/coal gangue ratios of 2.5:5 g, 5:5 g, and 10:5 g showed stable removal efficiencies of malachite green, crystal violet, methylene blue, and methyl violet at 95.17–97.61%, 91.03–92.60%, 79.47–84.16%, and 76.62–81.33%, respectively. Compared to coal gangue, Ca-CG prepared under different Ca:CG conditions exhibited a significant increase in dye removal rates. This enhancement can be attributed to the involvement of calcium in the dye adsorption process. Additionally, the optimized Ca-CG prepared under the mentioned conditions showed improvements in the removal efficiencies of malachite green, crystal violet, methylene blue, and methyl violet at 200 mg/L by 52.37%, 53.11%, 49.07%, and 51.18%, respectively. Hamid Z. et al. [24] synthesized a new type of magnesium–aluminum hydroxide, which achieved a maximum adsorption removal rate of 98% for malachite green at 46 mg/L. An S. et al. [20] prepared a novel calcium-based adsorbent CaFe_2_O_4_, which only achieved a maximum removal rate of 30.4% for crystal violet at 24.5 mg/L. Amoh et al. [25] prepared KOH-WTP using alkali-modified waste carbon powder, achieving an adsorption removal rate of 80% for methylene blue at 10 mg/L within 1 h. Sanjay et al. [26] developed a magnetic Fe_3_O_4_-coir, which achieved a maximum removal rate of >98% for methyl violet dye at 100 mg/L. Compared to the aforementioned magnesium–aluminum hydroxide, CaFe_2_O_4_, KOH-WTP, and Fe_3_O_4_-coir adsorbents, the Ca-CG prepared in this study still exhibited good removal efficiency for high concentrations of dyes. Moreover, Ca-CG showed good adsorption performance for multiple dyes, expanding its application range in dye wastewater treatment. However, compared to the magnetic Fe_3_O_4_-coir, although Ca-CG had a high dye adsorption efficiency, it lacked magnetism, making the recovery and reuse process more complex than Fe_3_O_4_-coir. By comparing the dye removal rates of Ca-CG prepared under different Ca:CG conditions, it was found that the Ca-CG prepared with a CaCl_2_/coal gangue ratio of 2.5:5 g exhibited the highest dye removal rate. Interestingly, as the CaCl_2_/coal gangue ratio increased (5 g, 5:5 g, 10:5 g), there was a slight decrease in the dye removal rates by Ca-CG. This is mainly because the calcium content far exceeds that of coal gangue, with a large amount of calcium occupying the active sites on the coal gangue surface, reducing the adsorption efficiency of coal gangue for dyes. However, the impact of this variation in the Ca:CG ratio on dye removal was limited. Therefore, considering drug savings and cost reduction, subsequent experiments selected a CaCl_2_/coal gangue ratio of 2.5:5 g to prepare Ca-CG.

### 2.2. Batch Experiments

**Figure 2:**
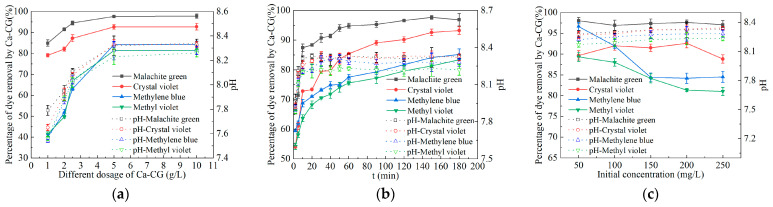
Batch experiments of different factors affecting the adsorption of malachite green, methylene blue, crystal violet and methyl violet by Ca-CG. (**a**) Influence of Ca-CG dosage (dye initial concentration: 200 mg/L, 298.15 K, 150 r/min, 150 min). (**b**) Influence of reaction time (dye initial concentration: 200 mg/L, Ca-CG dosage: 5 g/L, 298.15 K, 150 r/min). (**c**) The effect of different initial dye concentrations (Ca-CG dosage 5 g/L, 298.15 K, 150 rpm, 150 min).

Under different Ca-CG dosage (1, 2, 2.5, 5, 10 g/L), different reaction time (2, 5, 10, 20, 30, 40, 50, 60, 90, 120, 150, 180 min) and different initial dye concentration (50, 100, 150, 200, 250 mg/L), the adsorption removal rates of malachite green, methylene blue, crystal violet and methyl violet by Ca-CG are shown in Figure 2.

It can be seen from Figure 2a that the adsorption of malachite green, methylene blue, crystal violet and methyl violet by Ca-CG increased first and then stabilized with the increase in Ca-CG dosage. When the dosage of Ca-CG increased from 1 g/L to 5 g/L, the removal percentage of malachite green, crystal violet, methylene blue and methyl violet by Ca-CG increased from 84.91%, 79.12%, 40.11% and 41.49% to 97.61%, 92.60%, 84.16% and 81.33%, respectively. When the dosage of Ca-CG increased from 5 g/L to 10 g/L, the percentage of adsorption removal of malachite green, methylene blue, crystal violet and methyl violet by Ca-CG remained basically stable. This showed that when the Ca-CG dosage was 1–5 g/L, the Ca-CG dosage was the main factor affecting the dye adsorption effect. However, when Ca-CG exceeds 5 g/L, the dosage of Ca-CG will not affect the adsorption effect of Ca-CG on dyes. It can be seen from Figure 2b that the adsorption of malachite green, methylene blue, crystal violet and methyl violet by Ca-CG increased first and then stabilized with time pass by. When the adsorption time was 180 min, the adsorption removal percentage of malachite green, crystal violet, methylene blue and methyl violet by Ca-CG reached 96.85%, 93.19%, 85.18% and 83.36%, respectively. It can be seen from Figure 2c that the adsorption of malachite green by Ca-CG remained stable with the increase in malachite green concentration. The adsorption of crystal violet by Ca-CG increased first and then decreased with the increase in crystal violet concentration, and the maximum adsorption removal percentage was 92.60%. The adsorption of methylene blue and methyl violet by Ca-CG decreased first and then stabilized with the increase in dye concentration, and finally stabilized at about 85% and 80%. In summary, the adsorption performance of Ca-CG for four dyes is as follows: malachite green > crystal violet > methylene blue > methyl violet. The dosage of Ca-CG had a great influence on the adsorption of four dyes by Ca-CG. Within a short period (0–60 min), Ca-CG exhibited rapid adsorption for four dyes. As time progressed, the adsorption efficiency gradually stabilized. The initial concentration of methylene blue and methyl violet had a significant impact on their adsorption by Ca-CG.

From Figure 2a–c, it can be observed that under different conditions such as Ca-CG dosage, reaction time, and initial dye concentration, the pH of the solution after adsorption of malachite green, methylene blue, crystal violet, and methyl violet by Ca-CG increased from 7 to 8.22–8.34. This indicates that Ca-CG releases alkalinity in dye solutions, raising the pH of the solution. It has been reported that as pH increases, the negative charge on the adsorbent surface also increases, enhancing the electrostatic interaction between the adsorbent and the dye, thereby increasing the adsorption capacity for the dye [27]. Furthermore, under alkaline conditions, hydroxide ions may also form co-adsorption with dye molecules, making the decolorization of dye solutions easier to accomplish [28].

### 2.3. Adsorption Kinetics

**Figure 3:**
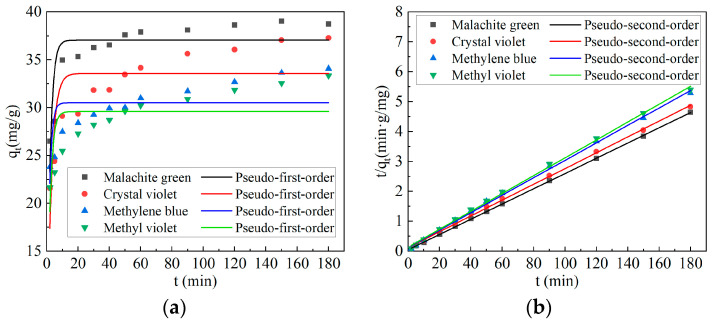
The adsorption kinetics fitting curve of malachite green, methylene blue, crystal violet and methyl violet on Ca-CG (dye initial concentration: 200 mg/L, Ca-CG dosage: 5 g/L, 298.15 K, 150 r/min). (**a**) First-order kinetic fitting curve. (**b**) Second-order kinetic fitting curve.

The fitting results of adsorption kinetics of malachite green, methylene blue, crystal violet and methyl violet by Ca-CG are shown in Figure 3 and Table 1.

From Figure 3a,b and Table 1, it can be seen that when Ca-CG adsorbed malachite green, methylene blue, crystal violet, and methyl violet, the *R*^2^ of the second-order kinetic fitting was greater than the *R*^2^ of the first-order kinetic fitting. It showed that the adsorption of Ca-CG on malachite green, methyl blue, crystal violet and methyl violet conformed to the second-order kinetic fitting model. The fitting equation of adsorption kinetics of malachite green by Ca-CG was *t*/*q_t_* = 0.02549*t* + 0.04982, *R*^2^ = 0.99987, *R*^2^ = 0.95363. The adsorption kinetics fitting equation of Ca-CG to methylene blue was: *t*/*q_t_* = 0.02918*t* + 0.12329, *R*^2^ = 0.99846. The adsorption kinetics fitting equation of Ca-CG to crystal violet was: *t*/*q_t_* = 0.0264*t* + 0.1268, *R*^2^ = 0.99892. The fitting equation of adsorption kinetics of methyl violet by Ca-CG was: *t*/*q_t_* = 0.02988*t* + 0.13615, *R*^2^ = 0.99849. The above research showed that the adsorption of malachite green, methylene blue, crystal violet and methyl violet by Ca-CG was mainly a chemical reaction.

### 2.4. Adsorption Isotherm and Adsorption Thermodynamics

**Figure 4:**
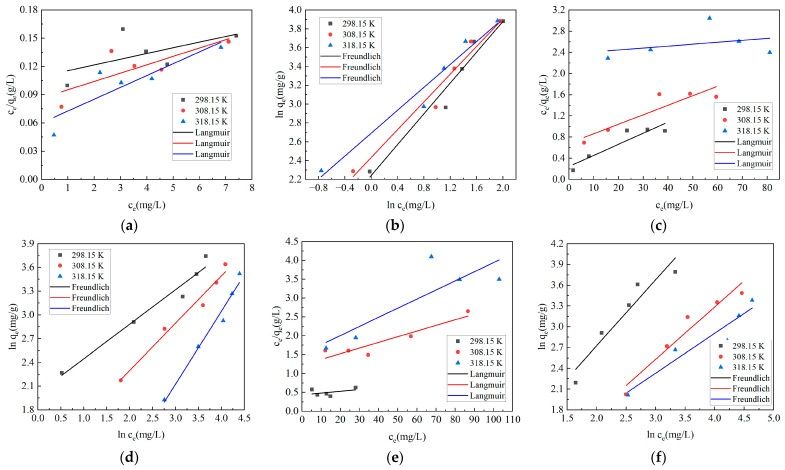

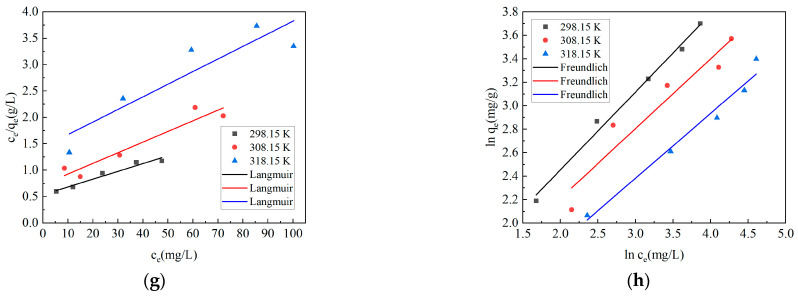
The isotherm fitting curves of malachite green, methylene blue, crystal violet and methyl violet adsorbed by Ca-CG (Ca-CG dosage: 5 g/L, 150 r/min, 150 min). (**a**) Langmuir fitting of Ca-CG adsorption of malachite green. (**b**) Freundlich fitting of Ca-CG adsorption of malachite green. (**c**) Langmuir fitting of Ca-CG adsorption of methylene blue. (**d**) Freundlich fitting of Ca-CG adsorption of methylene blue. (**e**) Langmuir fitting of Ca-CG adsorption of crystal violet. (**f**) Freundlich fitting of adsorption of crystal violet by Ca-CG. (**g**) Langmuir fitting of Ca-CG adsorption of methyl violet. (**h**) Freundlich fitting of Ca-CG adsorption of methyl violet.

At 298.15 K, 308.15 K and 318.15 K, the adsorption isotherms of malachite green, methylene blue, crystal violet, and methyl violet by Ca-CG are shown in Figure 4 and Table 2.

The adsorption isotherm can indicate the distribution of adsorbate molecules on the adsorbent in the adsorption equilibrium state, which has an important reference value for the analysis of the adsorption system. From Figure 4 and Table 2, it can be seen that when Ca-CG adsorbed malachite green, methylene blue, crystal violet, and methyl violet, the *R*^2^ fitted by the Freundlich model was greater than the *R*^2^ fitted by the Langmuir model. It showed that the adsorption of malachite green, methylene blue, crystal violet and methyl violet by Ca-CG conformed to the Freundlich model. This also showed that the adsorption of malachite green, methylene blue, crystal violet and methyl violet by Ca-CG was mainly multi-molecular layer adsorption. When 1/*n* was between 0 and 1, it indicated that it was easy to adsorb, and over 2 means that it is difficult to adsorb. When the values of 1/*n* for malachite green, methylene blue, crystal violet, and methyl violet adsorbed by Ca-CG were between 0 and 1, it indicated that Ca-CG had a strong affinity for these dyes.

From the adsorption thermodynamic parameters Δ*G*, Δ*H* and Δ*S* in Table 3, it can be seen that when the reaction temperatures were 298.15 K, 308.15 K and 318.15 K, the thermodynamic parameters Δ*G* of Ca-CG adsorption malachite green, methylene blue, crystal violet and methyl violet were all negative, indicating that the adsorption reaction can be carried out spontaneously. At the same time, the higher the temperature *T*, the smaller the Δ*G*, indicating that the spontaneous reaction was easier, that is, the spontaneous reaction of Ca-CG-adsorbing dyes was easier. The enthalpy change Δ*H* in the reaction process was negative, indicating that the adsorption of malachite green, methylene blue, crystal violet and methyl violet by OCa-CG was an exothermic process. The positive value of ∆*S* indicated that the adsorption of malachite green, methylene blue, crystal violet and methyl violet by Ca-CG was an entropy increase process, and the degree of chaos and disorder at the solid–liquid interface increased during the adsorption process [29]. Δ*S* was very small, indicating that the structure of the adsorbent Ca-CG only underwent slight changes [30].

### 2.5. XRF, FTIR, XRD and SEM Analysis

The major chemical compositions of coal gangue (CG) and Ca-CG were detected using the Shimadzu XRF-1800 X-ray fluorescence spectrometer (Shimadzu, Kyoto, Japan), and the results are presented in Table 4. Coal gangue primarily consists of SiO_2_, Al_2_O_3_, Fe_2_O_3_, MgO, K_2_O, P_2_O_5_, among others. Elements such as Si, Al, Fe, Mg, K, and P are the main constituents of minerals like quartz, berlinite, lipscombite, kaolinite, etc., found in coal gangue. The basic composition and content of coal gangue and Ca-CG are quite similar. Notably, the CaO content in Ca-CG increased from 1.87 to 3.84 compared to coal gangue, indicating successful loading of Ca onto the surface of coal gangue.

**Figure 5:**
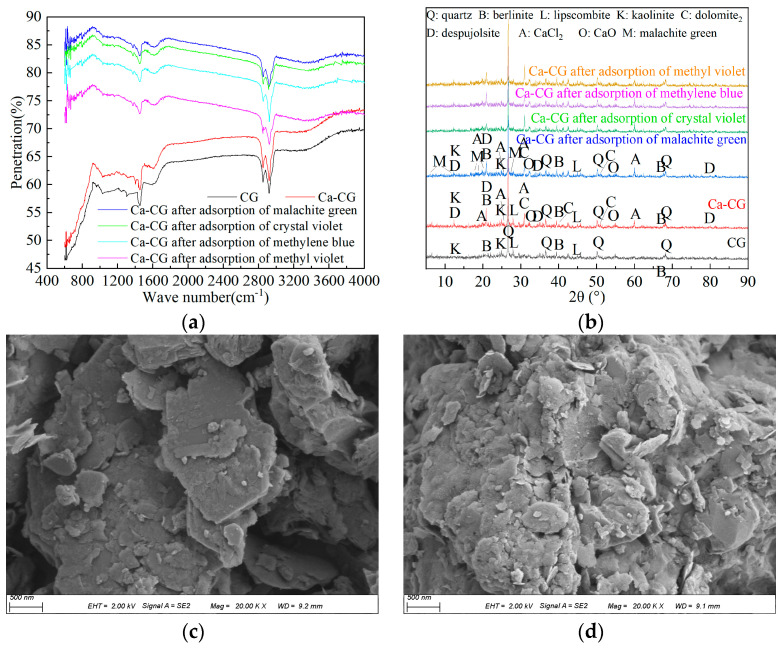
The FTIR and XRD graphs before and after Ca-CG adsorption of malachite green and other dyes. (**a**) FTIR before and after Ca-CG adsorption of dyes. (**b**) XRD before and after Ca-CG adsorption of dyes (**c**) SEM of Ca-CG. (**d**) SEM after adsorption of malachite green by Ca-CG.

From Figure 5a, it can be seen that the tensile vibration bands of Ca-CG at 1430 cm^−1^ and 668 cm^−1^ were related to the existence of Ca-O [31]. This showed that Ca was successfully loaded on coal gangue, forming a calcium-based modified coal gangue material. These peaks changed after the adsorption of malachite green by Ca-CG, indicating that Ca played an important role in the adsorption of malachite green. Interestingly, new functional groups appeared after the adsorption of malachite green by Ca-CG. For example, the benzene ring C-H out-of-plane bending vibration absorption peak appeared at 829 cm^−1^. The absorption peak of C-C skeleton stretching vibration appeared at 908 cm^−1^. The vibration absorption peak of the double carbon structure appeared at 1174 cm^−1^. The -C-N stretching vibration peak connected to the benzene ring appeared at 1370 cm^−1^. The antisymmetric deformation vibration absorption peak of -CH_3_ appeared at 1443 cm^−1^. The C=C stretching vibration peak of the olefin appeared at 1586 cm^−1^. The C=C stretching vibration peaks of the benzene ring appeared at 1614 and 1474 cm^−1^. The absorption peak of -C-N stretching vibration appeared at 1695 cm^−1^. The above functional groups were the characteristic functional groups of malachite green, and their appearance showed that malachite green was adsorbed on the surface of the adsorbent by Ca-CG. At the same time, the FTIR characteristic absorption peaks in the range of 600–800 cm^−1^ changed significantly, indicating that malachite green changed the surface functional groups of Ca-CG. The alteration of the mentioned functional groups suggested that Ca-CG can effectively adsorb malachite green dye, and the adsorption involved a chemical reaction that modified the functional groups on the surface of Ca-CG. It can be seen from Figure 5b that Ca-CG mainly contained quartz, berlinite, lipscombite, kaolinite, dolomite, despujolsite, CaCl_2_, CaO and other crystal structures. Among them, dolomite, despujolsite, CaCl_2_, CaO and other calcium-containing substances are mainly formed when Ca-CG is prepared, and Ca changes the mineral composition on the surface of coal gangue. The main crystal phases constituting coal gangue include quartz, berlinite, lipscombite, and kaolinite. The presence of diffraction peaks corresponding to calcium-containing substances such as dolomite, despujolsite, CaCl_2_, and CaO in Ca-CG indicates that Ca altered the mineral composition on the surface of coal gangue during Ca-CG preparation. The characteristic diffraction peaks of malachite green appeared near 6°, 13°, 18° and 27° after the adsorption of malachite green by Ca-CG. The characteristic diffraction peaks of lipscombite and dolomite disappeared after the adsorption of malachite green by Ca-CG, indicating that Ca-based participated in the adsorption of dyes by Ca-CG. After adsorbing malachite green, methylene blue, crystal violet, methyl violet, and other dyes, the characteristic diffraction peaks of Ca-CG did not change their positions significantly but only exhibited variations in peak heights. This indicates that Ca-CG did not alter the original internal crystal structure but rather experienced a decrease in crystallinity after the adsorption of these dyes. Particularly noteworthy is the incorporation of calcium ions from Ca-CG into the carbonate structure of coal gangue, forming dolomite. The disappearance of characteristic diffraction peaks of dolomite after Ca-CG adsorption of malachite green, methylene blue, crystal violet and methyl violet suggests the involvement of dolomite in dye adsorption. Dyes such as malachite green, methylene blue, crystal violet and methyl violet in the solution enter the interior structure of dolomite through ion exchange, thereby achieving the removal of dyes. The surface of Ca-CG was flat and smooth (Figure 5c). Comparing Figure 5c,d, it can be seen that after Ca-CG adsorbed malachite green, the surface of Ca-CG became rough, a large number of laminated structures appeared, and there were more flake particles. Combined with FTIR and XRD analysis, it can be seen that these flaky particle precipitates were formed by the surface precipitation reaction between malachite green and Ca-CG when Ca-CG adsorbed malachite green.

Coal gangue contains a large amount of inorganic cations (such as Na^+^, K^+^, Mg^2+^, etc.). During the process of calcium modification, calcium ions have strong exchangeability and can exchange with these cations in coal gangue. This allows more calcium ions to enter the interlayer of the coal gangue. XRF analysis showed that the CaO content in coal gangue was 1.87, while in Ca-CG it was 3.84, confirming that more calcium ions have entered the interlayer of coal gangue. As calcium ions enter the interlayer of coal gangue, the internal layers of coal gangue are gradually peeled off, increasing the specific surface area and exposing more negative charges. Ca-CG with a larger specific surface area had a stronger ability to adsorb dyes through electrostatic interactions. Additionally, the increased exposure of negative charges in Ca-CG enhanced the electrostatic interaction with cationic dyes such as malachite green, methylene blue, crystal violet and methyl violet. FTIR analysis revealed that compared to coal gangue, Ca-CG exhibited a significant presence of -COOH structures (such as stretching peaks of C=O, bending peaks of OH, and stretching peaks of C-O) in the range of 1300–1740 cm^−1^. Ca-CG also showed a significant presence of -COOR structures in the range of 1280–1100 cm^−1^. New free hydroxyl (-OH) groups were generated in Ca-CG between 3693 and 3614 cm^−1^, and stronger oxygen-containing functional groups like C-O/C-O-C were observed between 1093 and 1036 cm^−1^. These newly formed acidic oxygen-containing functional groups indicated that the calcium modification process exposed more carboxyl, ester, hydroxyl, and other structures in coal gangue, leading to more exposed negative charges in Ca-CG. The abundant negatively charged acidic oxygen-containing functional groups exhibited strong chelation with cationic dyes such as malachite green, methylene blue, crystal violet and methyl violet, achieving efficient adsorption and removal of dyes. Additionally, the FTIR analysis of adsorbed malachite green, methylene blue, crystal violet and methyl violet shows deviations, weakening, or disappearance of carboxyl, ester, hydroxyl, C-O/C-O-C, and other oxygen-containing functional groups in the ranges of 1300–1740 cm^−1^, 1280–1100 cm^−1^, 3693–3614 cm^−1^, and 1093–1036 cm^−1^, validating the presence of chelation between Ca-CG and dyes. In summary, the calcium modification process changed the interlayer structure of coal gangue through ion exchange, increased the specific surface area of coal gangue, and added more negative charges. This allowed Ca-CG to adsorb and remove cationic dyes such as malachite green, methylene blue, crystal violet and methyl violet through electrostatic and chelation interactions.

Malachite green, crystal violet, methylene blue, and methyl violet all contain benzene rings and -N(CH_3_)_2_ groups. The positively charged -N(CH_3_)_2_ group interacts electrostatically with the negatively charged functional groups on the surface of Ca-CG [32]. The deviations and weakening of oxygen-containing functional groups such as carboxyl, ester, hydroxyl, and C-O/C-O-C in the ranges of 1300–1740 cm^−1^, 1280–1100 cm^−1^, 3693–3614 cm^−1^, and 1093–1036 cm^−1^ in the FTIR analysis after dye adsorption indicate varying degrees of chelation between Ca-CG and the four dyes. The difference in chelation strength may be the reason for the varying adsorption effectiveness of Ca-CG for these dyes, with the order being malachite green > crystal violet > methylene blue > methyl violet.

## 3. Materials and Methods

### 3.1. Experimental Materials

The CaCl_2_ used in the test was produced by Tianjin Dengfeng Chemical Reagent Factory (Tianjin, China). Malachite green, methylene blue, crystal violet, methyl violet were produced by the Tianjin Fuchen Chemical Reagent company (Tianjin, China) limited. The chemicals used in the experiment were of analytical grade. The deionized water used in the experiment was prepared using an ultrapure water machine (YL-400BU model, Shenzhen Ereeran Water Treatment Equipment Co., Ltd., Shenzhen, China).

The preparation procedure of Ca-CG is as follows: Firstly, the coal gangue taken from a mining area in Fuxin City, Liaoning Province was crushed, and the coal gangue with a particle size of 100 mesh was screened. Secondly, CaCl_2_, coal gangue, and 200 mL of deionized water were added to a conical flask. The ratios of CaCl_2_ to coal gangue were 0:5 g, 2.5:5 g, 5:5 g, and 10:5 g, respectively, producing different Ca:CG ratios of Ca-CG. The conical bottle containing the mixture was placed in an ultrasonic water bath in a YM-0315 ultrasonic cleaning machine (Model YM-0315, Shenzhen Fangao Microelectronics Co., Ltd., Shenzhen, China) for 30 min. Then, the above-mixed solution was placed on a constant temperature magnetic stirrer (Model HJ-1, Changzhou Surui Instruments Co., Ltd., Changzhou, China) at 60 °C and stirred for 12 h. Then, the solid phase was separated after standing for 1 h, and it was dried to constant weight in a blast drying oven (Model GZX-9246MBE, Shanghai Boxun Medical Biological Instruments Co., Ltd., Shanghai, China) at 60 °C to obtain Ca-CG. Different Ca:CG ratios of Ca-CG were added according to the solid–liquid ratio of 5 g/L to solutions of malachite green, methylene blue, crystal violet, and methyl violet at concentrations of 200 mg/L and volumes of 200 mL. Under conditions of 298.15 K and 150 r/min oscillation, they were adsorbed for 150 min; then, we measured the remaining concentrations of malachite green, methylene blue, crystal violet, and methyl violet. We calculated the removal rates of malachite green, methylene blue, crystal violet, and methyl violet by different Ca:CG ratios of Ca-CG, and optimized the preparation parameters of Ca-CG.

### 3.2. Experimental Method

In order to explore the adsorption kinetics, adsorption isotherms and adsorption thermodynamics of malachite green, methylene blue, crystal violet and methyl violet by Ca-CG, a series of batch experiments were carried out. Using a spectrophotometer (Model 721, Shanghai Jinghe Analytical Instrument Co., Ltd., Shanghai, China), we detected the concentrations of malachite green, methylene blue, crystal violet, and methyl violet before and after adsorption experiments at wavelengths of 617 nm, 664 nm, 590 nm, and 584 nm, respectively. Batch experiments of Ca-CG-adsorbing malachite green, methylene blue, crystal violet, and methyl violet were conducted with all other experimental steps being identical except for the different adsorbed dyes. The following is an example of a Ca-CG adsorption malachite green experiment.

Batch experiments: A certain mass of Ca-CG was added to 200 mL of malachite green simulated wastewater with a specified initial concentration. Adsorption experiments were conducted at 298.15 K and 150 r/min for a certain duration. By varying the Ca-CG dosage (1, 2, 2.5, 5, 10 g/L), reaction time (2, 5, 10, 20, 30, 40, 50, 60, 90, 120, 150, 180 min), and initial malachite green concentration (50, 100, 150, 200, 250 mg/L), the effects of different factors on Ca-CG’s removal of malachite green were investigated. Three groups of parallel experiments were set up in all experiments to detect the remaining malachite green concentration in the solution. According to the results of parallel experiments, the removal rate (*η*, %) of malachite green by Ca-CG was calculated. We took the average and standard deviation (error bars) of parallel experiments and plotted the data. The experimental steps for Ca-CG adsorption of methylene blue, crystal violet, and methyl violet were the same as described above.

(1) $$\eta =\frac{C_{0}-C_{e}}{C_{0}}\times 100$$

Adsorption kinetics experiment steps: We added 1 g of Ca-CG to a conical flask containing 200 mL of simulated wastewater with a concentration of 200 mg/L of malachite green. The reaction was carried out at 298.15 K and 150 r/min. At reaction times of 2, 5, 10, 20, 30, 40, 50, 60, 90, 120, 150, and 180 min, respectively, we collected water samples and measure the remaining concentration of malachite green in the solution. The experiment included three sets of parallel tests. The adsorption capacity (*q_e_*, mg/g) of malachite green by Ca-CG under different reaction time conditions was calculated. We took the average and standard deviation of parallel experiments (error bar values) and plotted the data graphically. The results were fitted by the Lagergren first-order kinetic equation (Formula (3)) and the Lagergren second-order kinetic equation (Formula (4)) to explore the adsorption kinetics of malachite green by Ca-CG. The experimental steps for Ca-CG adsorption of methylene blue, crystal violet, and methyl violet were the same as described above.

(2) $$q_{e}=\frac{{V\times (C}_{0}-C_{e})}{m}$$

(3) $$q_{t}=q_{e}\times (1-exp(-k_{1}\times t))$$

(4) $$\frac{t}{q_{t}}=\frac{1}{k_{2}q_{e}^{2}}+\frac{t}{q_{e}}$$

In the equation, *C*_0_ (mg/L) and *C_e_* (mg/L) are the initial concentration and equilibrium concentration of malachite green, respectively. *q_e_* (mg/g) is the adsorption capacity of Ca-CG for malachite green. *V* (L) is the volume of malachite green solution, and *m* (g) is the mass of adsorbent Ca-CG. *q_t_* (mg/g) is the adsorption capacity of Ca-CG to malachite green at *t* (min). *k*_1_ (min^−1^) and *k*_2_ (mg/(g·min)) are the adsorption rate constants of the Lagergren first-order kinetic model and the Lagergren second-order kinetic model, respectively.

The adsorption isotherm and adsorption thermodynamics experimental steps: We added 1 g of Ca-CG to a conical flask containing 200 mL of simulated wastewater with malachite green at concentrations of 50, 100, 150, 200, and 250 mg/L. We placed the flasks under conditions of 298.15 K, 308.15 K, and 318.15 K, and oscillated them at 150 r/min for 150 min; three parallel tests were performed for each test. The remaining malachite green concentration in the solution was detected. According to the results of parallel experiments, the adsorption capacity (*q_e_*, mg/g) of malachite green by Ca-CG under different temperature and concentration conditions was calculated. We took the average and standard deviation of parallel experiments (error bar values) and plotted the data graphically. Langmuir (Formula (5)) and Freundlich (Formula (6)) adsorption isotherm formulas were used to fit the results to explore the adsorption isotherm of malachite green by Ca-CG. The Gibbs free energy ∆*G* (kJ/mol), enthalpy change ∆*H* (kJ/mol) and entropy change ∆*S* (kJ/(mol·K)) were determined according to Formulas (7)–(9). The experimental steps for Ca-CG adsorption of methylene blue, crystal violet, and methyl violet were the same as described above.

(5) $$\frac{C_{e}}{q_{e}}=\frac{1}{q_{m}K_{L}}+\frac{C_{e}}{q_{m}}$$

(6) $$lnq_{e}=lnK_{F}+\frac{1}{n}lnC_{e}$$

(7) $$\Delta G=-RT\ln K_{c}$$

(8) $$K_{c}=\frac{q_{e}}{C_{e}}$$

(9) ΔG=ΔH−TΔS

In the formula, *q_e_* and *q_m_* (mg/g) represent the adsorption capacity of Ca-CG for malachite green at reaction equilibrium and adsorption saturation, respectively. *K_L_* (L/mg) and *K_F_* (mg^(1−1/*n*)^·L^1/*n*^·g^−1^) are the adsorption constants of Langmuir model and Freundlich model, respectively. *n* is the Freundlich model adsorption strength constant. *K_C_* (L/g) is the adsorption equilibrium coefficient of malachite green adsorbed by Ca-CG. *R* is usually 8.3145 J/(mol·K); *t* (K) is the reaction temperature.

XRF, FTIR, XRD and SEM detection methods: We took coal gangue and Ca-CG for XRF analysis using the XRF-1800 model from Shimadzu Corporation, Shimadzu, Kyoto, Japan, to analyze their main chemical compositions. We took coal gangue, Ca-CG, and Ca-CG with adsorbed dyes for analysis. Ca-CG and Ca-CG adsorbed malachite green were dried to constant weight at 30–35 °C. The functional groups of Ca-CG before and after adsorption were analyzed by Nicolet iS5 FTIR (Thermo Fisher Scientific, Waltham, MA, USA). The crystal structure of Ca-CG before and after adsorption was analyzed by Japanese Rigaku Smart Lab 9 XRD (Rigaku, Tokyo, Japan). The surface morphology of Ca-CG before and after adsorption was observed using a Germany Zeiss 4 Sigma 500 SEM (Germany Zeiss, Oberkochen, Germany).

## 4. Conclusions

In this paper, a new dye adsorbent Ca-CG was prepared by the ultrasonic method using coal gangue and CaCl_2_ as raw materials. The adsorption performance of Ca-CG for four dyes is as follows: malachite green > crystal violet > methylene blue > methyl violet. When the dosage of Ca-CG was 1–5 g/L, the dosage of Ca-CG was the main factor affecting the adsorption effect of dyes. In a short time (0–60 min), Ca-CG adsorbed four dyes faster, and the adsorption effect gradually tended to be stable with the extension of time. The initial concentration of methylene blue and methyl violet had a significant impact on their adsorption by Ca-CG. The adsorption of malachite green, methylene blue, crystal violet, and methyl violet by Ca-CG all conformed to a second-order kinetic fitting model, with adsorption primarily driven by chemical reactions. Ca-CG’s adsorption for the four dyes also fitted the Freundlich model, indicating multilayer adsorption as the main mechanism, which was easily achieved. Lipscombite, dolomite, and other calcium-based materials are crucial for the adsorption of malachite green by Ca-CG, with surface precipitation, electrostatic action, and chelation reaction being the main mechanisms. The prepared Ca-CG can serve as an economical and efficient adsorbent for removing dyes from water, providing a feasible method for the resource utilization of coal gangue. The dye-adsorbed Ca-CG can be regenerated through desorption pathways and reused in the field of water pollution control. However, further exploration is needed in desorption methods and the field of regeneration and reuse.

## Figures and Tables

**Table 1:** Kinetic fitting data of Ca-CG-adsorbing malachite green, methylene blue, crystal violet and methyl violet.

Adsorption Kinetics Model	Parameter	Dye			
		Malachite Green	Methylene Blue	Crystal Violet	Methyl Violet
Pseudo-first-order	*q_e_* (mg/g)	37.05617	30.521	33.57172	29.60306
	*k*_1_ (min^−1^)	0.49497	0.64269	0.36501	0.52046
	*R* ^2^	0.68002	0.41459	0.61455	0.471
Pseudo-second-order	*q_e_* (mg/g)	39.23107	34.27005	37.87879	33.46720
	*k*_2_ (mg/(g·min))	0.01304	0.00691	0.00550	0.00656
	*R* ^2^	0.99987	0.99846	0.99892	0.99849

**Table 2:** Isothermal fitting data of malachite green, methylene blue, crystal violet and methyl violet adsorbed by Ca-CG.

Type of Dyes	*T* (K)	Langmuir Model			Freundlich Model		
		*q_m_* (mg/g)	*K_L_* (L/mg)	*R* ^2^	*K_F_* (mg^(1−1/*n*)^·L^1/*n*^·g^−1^)	*n*	*R* ^2^
Malachite green	298.15	165.01650	0.05531	0.35049	9.44089	1.22424	0.95946
	308.15	112.86682	0.10250	0.61541	11.41799	1.35811	0.96814
	318.15	79.68127	0.20847	0.76412	14.78318	1.64493	0.96388
Methylene blue	298.15	47.59638	0.08607	0.86264	7.46937	2.29216	0.96673
	308.15	55.71031	0.02622	0.83852	3.01413	1.67193	0.97531
	318.15	274.72527	0.00153	0.10702	0.52860	1.08516	0.97551
Crystal violet	298.15	207.90021	0.01106	0.18798	2.28022	1.05048	0.91143
	308.15	66.71114	0.01224	0.86068	1.30412	1.32366	0.94166
	318.15	41.28819	0.01592	0.72858	1.83543	1.73986	0.92135
Methyl violet	298.15	67.88866	0.02738	0.95627	3.08892	1.50707	0.99065
	308.15	49.60317	0.02770	0.91203	2.78250	1.68277	0.91439
	318.15	41.91115	0.01661	0.91301	2.06630	1.81018	0.97279

**Table 3:** Thermodynamic parameters of Ca-CG-adsorbing dyes.

Type of Dyes	Δ*G* (kJ/mol)			Δ*H* (kJ/mol)	Δ*S* (kJ/(mol·K))
	298.15 K	308.15 K	318.15 K		
Malachite green	−4.19610	−4.33684	−4.47758	−2.94918 × 10^−9^	0.01407
Methylene blue	−0.60784	−0.62823	−0.64861	−1.05108 × 10^−8^	0.00204
Crystal violet	−2.62222	−2.71017	−2.79812	−4.545 × 10^−9^	0.00879
Methyl violet	−0.01334	−0.01379	−0.01424	1.2415 × 10^−8^	4.47441 × 10^−5^

**Table 4:** Major chemical compositions (%) of coal gangue and Ca-CG.

Components	SiO_2_	Al_2_O_3_	Fe_2_O_3_	MgO	K_2_O	CaO	Na_2_O	TiO_2_	MnO	P_2_O_5_	SO_3_	CO_2_	Other
Coal gangue	60.81	18.47	4.98	2.95	2.19	1.87	1.54	0.81	0.08	2.35	0.28	2.56	1.11
Ca-CG	59.29	17.71	4.95	3.04	2.18	3.84	1.58	0.81	0.08	2.37	0.33	2.94	0.88

## Data Availability

All the data have been included in the study.
